# Development of 1-*N*-^11^C-Methyl-l- and -d-Tryptophan for pharmacokinetic imaging of the immune checkpoint inhibitor 1-Methyl-Tryptophan

**DOI:** 10.1038/srep16417

**Published:** 2015-11-10

**Authors:** Lin Xie, Jun Maeda, Katsushi Kumata, Joji Yui, Yiding Zhang, Akiko Hatori, Nobuki Nengaki, Hidekatsu Wakizaka, Masayuki Fujinaga, Tomoteru Yamasaki, Yoko Shimoda, Makoto Higuchi, Tetsuya Suhara, Feng Wang, Ming-Rong Zhang

**Affiliations:** 1 Molecular Imaging Center, National Institute of Radiological Sciences, 4-9-1 Anagawa, Inage-ku, Chiba 263-8555, Japan; 2 Department of Nuclear Medicine, Nanjing Hospital, Affiliated to Nanjing Medical University, 68 Chanle Road, Nanjing 210006, China

## Abstract

1-Methyl-tryptophan (1MTrp) is known as a specific inhibitor targeting the immune- checkpoint protein indoleamine-2,3-dioxygenase, in two stereoisomers of levorotary (l) and dextrorotary (d). A long-standing debate exists in immunology and oncology: which stereoisomer has the potential of antitumor immunotherapy. Herein, we developed two novel radioprobes, 1-*N*-^11^C-methyl-l- and -d-tryptophan (^11^C-l-1MTrp and ^11^C-d-1MTrp), without modifying the chemical structures of the two isomers, and investigated their utility for pharmacokinetic imaging of the whole body. ^11^C-l-1MTrp and ^11^C-d-1MTrp were synthesized rapidly with radiochemical yields of 47 ± 6.3% (decay-corrected, based on ^11^C-CO_2_), a radiochemical purity of >98%, specific activity of 47–130 GBq/μmol, and high enantiomeric purity. PET/CT imaging in rats revealed that for ^11^C-l-1MTrp, the highest distribution of radioactivity was observed in the pancreas, while for ^11^C-D-1MTrp, it was observed in the kidney. *Ex vivo* biodistribution confirmed the PET/CT results, indicating the differences in pharmacokinetics between the two isomers. Both ^11^C-l-1MTrp and ^11^C-d-1MTrp are therefore useful PET probes for delineating the distribution and action of the checkpoint inhibitor 1MTrp *in vivo*. This study represents the first step toward using whole-body and real-time insight to disentangle the antitumor potential of the two stereoisomers of 1MTrp, and it can facilitate the development of 1MTrp immunotherapy.

Advances in molecular imaging via positron-emission tomography (PET) have brought about a new type of precision pharmacology^1^. Using a radioisotope-labeled drug molecule, called a PET probe, allows tracking and quantification of the *in vivo* distribution and action of the potential drug molecule in a rapid, reproducible, and noninvasive manner. This type of pharmacokinetic imaging is now increasingly being used to shed light on several aspects of the development of new therapeutic strategies such as understanding relationships between the drug molecule and target tissue, evaluating the correlation of drug concentrations with effector function, identifying pharmacological differentiation among closely related drug molecules, deciding on an optimal dose and schedule, and stratifying the clinical population^1^,^2^,^3^,^4^.

1-Methyl-tryptophan (1MTrp) is an indole-containing inhibitor of indoleamine-2,3-dioxygenase (IDO), a rate-limiting enzyme in the catabolism of tryptophan^5^,^6^. It has been recently found that IDO joins the CTLA4 and PD1 group known as immune-checkpoint proteins involved in creating immunosuppressive microenvironment to mediate tumor immune escape^5^,^6^,^7^,^8^. By locally depleting tryptophan and generating pro-apoptotic metabolites, IDO induces anergy and death of effector T cells, the most powerful weapon of the immune system^9^. The tryptophan analogue 1MTrp, by competitively blocking the activity of IDO in tumors and tumor-infiltrating myeloid cells, shows superior antitumor potential^5^,^6^,^10^,^11^,^12^. Moreover, compared to the current antibody-based checkpoint inhibitors such as the anti-CTLA4 agent ipilimumab and the anti-PD1 agent nivolumab, the small-molecule 1MTrp is an affordable compound with good oral bioavailability and no immune-related toxicities that occurred by the antibody-based checkpoint inhibitors^7^,^13^. For these reasons, 1MTrp is currently of great interest in the field of immunotherapeutic manipulation.

Indeed, 1MTrp is being developed as an immunomodulatory anticancer agent for clinical trials. However, 1MTrp exists as two stereoisomers: levorotary (l) and dextrorotary (d); most preclinical studies have employed a racemic mixture^5^,^6^, sparking a long-standing debate in the fields of immunology and oncology about which stereoisomer has the potential of checkpoint blockade^14^,^15^,^16^. When the capacity of the two isomers for inhibiting IDO was analyzed separately, contradictory results have also been found. In *in vitro* studies, the l isomer was a strong inhibitor of IDO (*K*i = 19 μM), whereas the d isomer was much weaker effective (*K*i > 100 μM); however, in *in vivo* studies, the d isomer showed superior antitumor activity and lost its immunomodulatory effect in IDO-deficient mice. Therefore, d-1MTrp was selected as the lead IDO inhibitor in phase 1 clinical trials^14^. These strikingly conflicting findings are to some extent ascribed to that the two isomers of 1MTrp may have different pharmacokinetics, biological effects and bioavailability *in vivo*.

As the first step in distinguishing the antitumor potential and exploring the possibility of pharmacokinetic imaging in development of immunotherapy, we here developed 1-*N*-^11^C-methyl-l- and -d-tryptophan (^11^C-l-1MTrp and ^11^C-d-1MTrp) as individual PET probes. Using these two ^11^C-labelled isomers, without changing their chemical structures and properties, we delineated and compared their pharmacokinetics *in vivo* by dynamic PET/CT imaging. This study provides the first whole-body and real-time pharmacokinetic platform for disentangling the potential of the l and d isomers of 1MTrp. Moreover, it represents an initial step in pharmacokinetic imaging for the development of immunotherapy.

## Results

### Radiosynthesis of ^11^C-l-1MTrp and ^11^C-d-1MTrp

**Figure 1:**
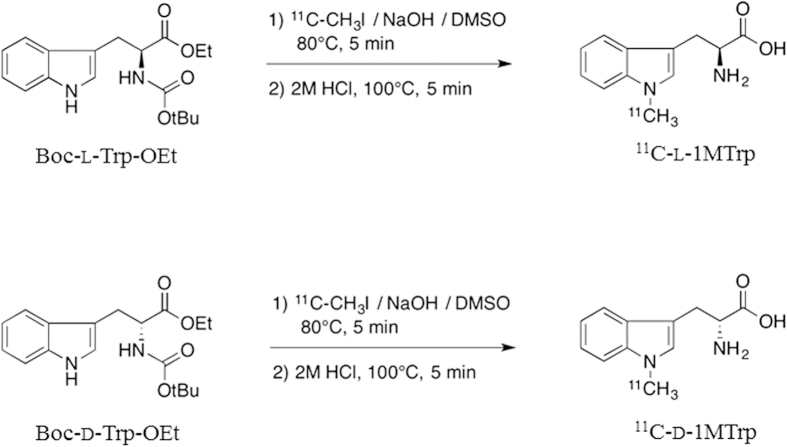
Chemical scheme for radiosynthesis of ^11^C- l-1MTrp and ^11^C-d-1MTrp.

^11^C-l-1MTrp and ^11^C-d-1MTrp were synthesized by *N*-methylation of the corresponding Boc-Trp-OEt precursor with ^11^C-Methyl iodide (^11^C-CH_3_I), using NaOH as a base (Fig. 1), followed by deprotection of the Boc and ethyl groups with HCl. After the reaction, high-performance liquid chromatography (HPLC) separation, and formulation, ^11^C-l-1MTrp and ^11^C-d-1MTrp with a sufficient amount of radioactivity was successfully obtained. At the end of synthesis (EOS), ^11^C-l-1MTrp and ^11^C-d-1MTrp of 2.6–3.7 GBq were obtained as injectable solutions by 40 ± 2 min after completing bombardment. The decay-corrected radiochemical yield of ^11^C-l-1MTrp and ^11^C-d-1MTrp based on ^11^C-carbon dioxide (^11^C-CO_2_) was 47 ± 6.3% (n = 80) and the specific activity was 47−130 GBq/μmol at EOS. The averaged radiochemical purity of ^11^C-l-1MTrp and ^11^C-d-1MTrp was 98 ± 2.3% and remained in excess of 95% after 90 min. In the final solutions, the enantiomeric purity of both ^11^C-l-1MTrp and ^11^C-d-1MTrp solutions exceeded 80% ee.

### *In vitro* stability of ^11^C-l-1MTrp and ^11^C-d-1MTrp

To determine the *in vitro* property of the two newly-developed radioprobes, we performed plasma stability studies by HPLC analysis at 37 °C for 90 min. Both ^11^C-l-1Mtrp and ^11^C-d-1Mtrp were highly stable in rat plasma. More than 95% of ^11^C-l-1Mtrp and ^11^C-d-1Mtrp remained intact and no detectable metabolites were found at least 90 min after plasma incubation *in vitro*.

### Whole-body pharmacokinetic imaging by PET/CT

**Figure 2:**
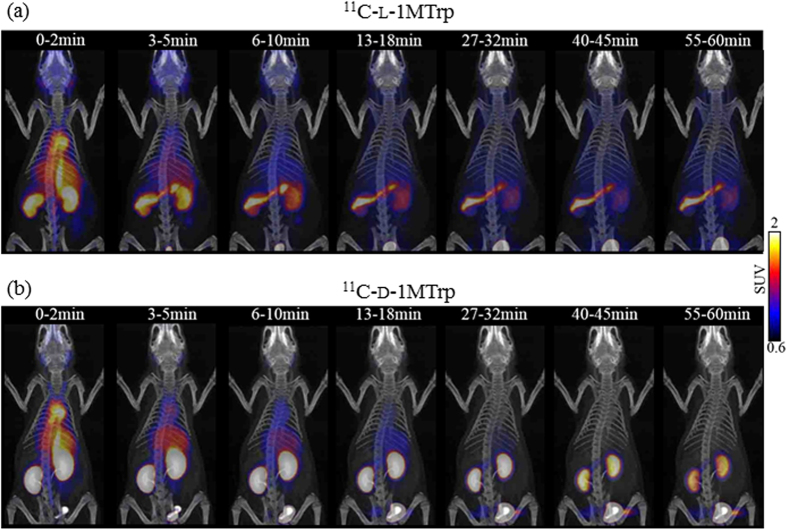
Representative pharmacokinetic images of the two isomers. (**a**) Three-dimensional PET/CT images at different time points after ^11^C-l-1MTrp injection. (**b**) Three-dimensional PET/CT images at different time points after ^11^C-d-1MTrp injection.

To visualize global pharmacokinetics of l-1MTrp and d-1MTrp *in vivo*, we performed dynamic PET/CT scans in rats from 0 to 60 min after ^11^C-l-1MTrp or ^11^C-d-1MTrp injection. Figure 2 exhibits typical pharmacokinetic images of the two isomers. At 2 min immediately after administration, ^11^C-l-1MTrp and ^11^C-d-1MTrp were similarly carried through the vena cava to the heart, and rapidly distributed throughout the whole body. Using PET/CT images, we clearly visualize that ^11^C-l-1MTrp was characterized by a continuous accumulation in the pancreas, with rapid washout from the blood and renal excretion, during the initial 3–18 min (Fig. 2a). Thereafter, the high uptake in the pancreas was constant, while low accumulation of radioactivity was seen in the brain, heart, lung, and other abdominal organs such as the liver, spleen, and intestine, resulting in excellent pancreas-to-background contrast images (Fig. 2a). On the other hand, ^11^C-d-1MTrp exhibited a different distribution pattern from ^11^C-l-1MTrp (Fig. 2b). The highest retention of ^11^C-d-1MTrp was observed in the kidneys, with rapid renal clearance, and negligible radioactivity was observed in the blood, brain, muscle, and abdominal tissues, including the pancreas.

**Figure 3:**
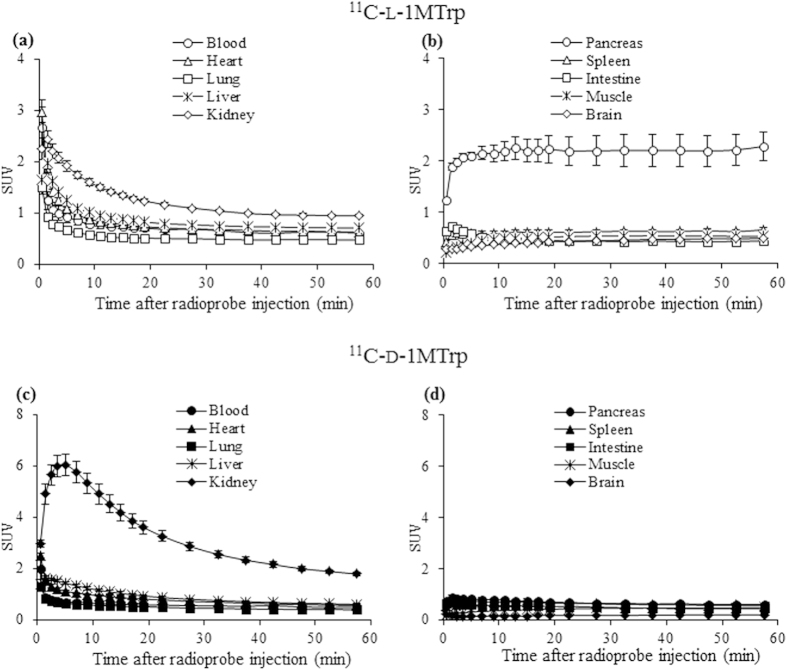
Temporal changes in the distribution of the two isomers throughout the body. (**a**) Time−activity curves (TACs) of ^11^C-l-1MTrp in the blood, heart, lungs, liver, and kidneys. (**b**) TACs of ^11^C-l-1MTrp in the pancreas, spleen, intestines, muscle, and brain. (**c**) TACs of ^11^C-d-1MTrp in the blood, heart, lungs, liver, and kidneys. (**d**) TACs of ^11^C-d-1MTrp in the pancreas, spleen, intestines, muscle, and brain. Data are expressed as means with SEM from four rats.

Next, to better understand the temporal distribution of the two isomers in various locations of the whole body, we quantified the radioactivity related to ^11^C-l-1MTrp and ^11^C-d-1MTrp over time in individual organs and tissues based on the dynamic PET scans. Time-activity curves (TACs) of ^11^C-l-1MTrp and ^11^C-d-1MTrp are shown in Fig. 3. During the initial phase after radioprobe injection, both ^11^C-l-1MTrp and ^11^C-d-1MTrp exhibited high radioactivity in the main organs (heart, lungs, liver, and kidneys), which was paralleled by intense radioactivity in the blood, reflecting the rich perfusion of these organs and tissues (Fig. 3a,c). ^11^C-l-1MTrp rapidly accumulated in the pancreas; reached a highest uptake in the pancreas (SUV 2.28 ± 0.28), higher in the kidney (0.94 ± 0.03), followed by the liver, spleen, heart, blood, muscle, lung, brain and intestine (0.43 ~ 0.70), at 60 min after injection (Fig. 3a,b).

**Figure 4:**
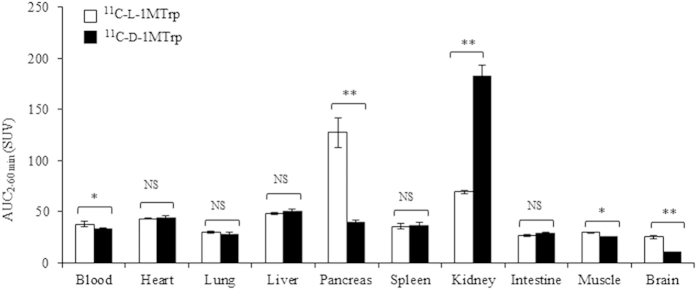
Comparison of the distribution of the two isomers *in vivo*. Area under time−activity curves (AUC_2–60min_) of PET with ^11^C-l-1MTrp and ^11^C-d-1MTrp. Distribution of the two isomers in individual organs and tissues were compared using Student’s *t*-test. Asterisks indicate statistical significance (*p < 0.05, **p < 0.01). NS, not significant. Data are expressed as means with SEM from four rats.

On the other hand, maximum accumulation of ^11^C-d-1MTrp was observed in the kidneys (SUV 6.03 ± 0.43) within 5 min after administration, after which it declined. At 60 min, ^11^C-d-1MTrp was highly retained only in the kidneys (SUV 1.79 ± 0.07), with very low radioactivity in the other organs (0.20−0.61; Fig. 3c,d). Renal elimination dominated the whole-body distribution of ^11^C-d-1MTrp in normal rats. Figure 4 summarizes the accumulation of the two isomers, which was quantified and represented as values of area under time-activity curves (AUC _2–60 min_) in individual organs and tissues.

Significant differences in the tissue distribution between the two isomers were validated in the blood, pancreas, kidneys, muscle, and brain (p < 0.05). These results indicated that, using PET with ^11^C-l-1MTrp and ^11^C-d-1MTrp, the *in vivo* behaviors of the two isomers can be visualized and quantified in a temporal−spatial pattern, and the different *in vivo* pharmacokinetics between l and d isomers of 1MTrp could be verified.

### *Ex vivo* biodistribution results

To confirm the *in vivo* pharmacokinetic imaging of the two isomers, the distribution of radioactivity in the main organs and tissues was measured at designated time points after injection of ^11^C-l-1MTrp (Table 1) and ^11^C-d-1MTrp (Table 2) in rats. Similar to the PET images, high radioactivity was verified in the main organs and tissues during the initial phase after ^11^C-l-1MTrp and ^11^C-d-1MTrp injection. For ^11^C-l-1MTrp, the highest uptake was observed in the pancreas (3.30% ± 0.12% ID/g at 5 min), while for ^11^C-d-1MTrp the highest accumulation (7.06% ± 0.57% ID/g at initial 1 min, followed by 6.21% ± 0.64% ID/g at 5 min) was observed in the kidneys. At 60 min after radioprobe injection, with the exception of the kidneys (l *vs.* d: 0.74 ± 0.04% ID/g *vs.* 1.16 ± 0.03% ID/g), the measured uptake of the l isomer (0.31−0.50% ID/g) was higher than for the d isomer (0.15−0.37% ID/g) in all organs and was the highest in the pancreas (l *vs.* d: 2.27 ± 0.05% ID/g *vs.* 1.03 ± 0.06% ID/g). Thus, the *ex vivo* biodistribution analysis confirmed the pharmacokinetic PET/CT images, and provided direct evidence of ^11^C-l-1MTrp and ^11^C-d-1MTrp accumulation in these organs and tissues, with a dose index.

### *Ex vivo* metabolite results

To validate the derivation of radioactivity into the relevant organs and tissues, we assessed the metabolites of both radiolabelled isomers in the blood, brain, and pancreas of rats. Radioactivity of more than 94% in the brain, pancreas, and blood was associated with l- and d-1MTrp (Table 3). Few radioactive metabolites were detected in these three tissues at 15 and 60 min after ^11^C-l-1MTrp and ^11^C-d-1MTrp injection. These results indicated that the radioactivity detected by PET were mainly due to the l-1MTrp- and d-1MTrp-bound radioisotopes, and not due to non-specific binding of the metabolites of these compounds.

## Discussion

In this study, we pioneered the idea of direct pharmacokinetic imaging in the immunotherapy field by developing novel PET probes derived from the checkpoint inhibitor 1MTrp, ^11^C-l-1MTrp and ^11^C-d-1MTrp. Using dynamic PET/CT, the behavior and action of the l and d stereoisomers of 1MTrp were temporal−spatially captured and compared across the body. This quantitative, imaging-based pharmacokinetic study represents the first step toward not only disentangling the checkpoint blockade potential of the two isomers of 1MTrp, but also forms the basis for pharmacokinetic imaging with a view of immunotherapy development.

The notable success of immune checkpoint inhibitors and their fast approval by the FDA for the treatment of solid and hematological malignancies have made immunotherapy a new treatment paradigm in oncology^7^,^17^,^18^,^19^,^20^. Unlike conventional chemotherapy or targeted therapy, which acts directly on the tumor, cancer immunotherapies exert their effects on the immune system by enhancing the cellular immune response, which results in changes in tumor burden or patient survival. Consequently, new challenges have emerged in immunotherapy trails, including stratification of the patient subpopulation, optimization of therapeutic strategies, and redefinition of response criteria^7^.

Precision pharmacology, which involves labeling drug molecules with positron-emitting radioisotopes, and then using PET to track and compare the *in vivo* behavior of the drug molecules, is an attractive approach to discovering novel therapeutic strategies owing to its excellent capacity for capturing drug molecules *in vivo* over time and represents an exciting innovation in drug development^1^. ^11^C and ^18^F are the most frequently used radioisotopes because they can label the molecule with no or minimal changes to the chemical structures or properties of the compounds and can provide strong imaging signals^2^. Considering the indole structure of 1MTrp, we designed a radiolabeling strategy in which Boc was used as an amine-protecting group and ethyl ester was used as a carboxyl-protecting group. By introduced ^11^C to the indole ring of l and d isomers, we synthesized ^11^C-l-1MTrp and ^11^C-d-1MTrp without modifying their chemical structures (Fig. 1). The two radioprobes were obtained successfully with sufficient radioactivity, high radiochemical yield, and reliable quality control, indicating that ^11^C-l-1MTrp and ^11^C-d-1MTrp can feasibly be produced and used for clinical evaluation.

1MTrp, with its superior antitumor effects and reduced toxicity, is particularly attractive for therapeutically manipulating antitumor immunity. However, obstacles standing in the developing way similar to the checkpoint antibodies, additional key knowledge gaps about the discrepancy of two stereoisomers during the *in vitro* and *in vivo* studies, throwing 1MTrp in a dilemma for further clinical development^5^,^6^,^14^,^15^. Directly tracking 1MTrp molecule can give us rationale and confidence to address the obstacles and gaps. Using ^11^C-l-1MTrp and ^11^C-d-1MTrp with dynamic PET/CT, we performed pharmacokinetic imaging of the two isomers of 1MTrp (Figs 2, 3 and 4), and confirmed that the obtained PET images were directly derived from the labeled l-1MTrp and d-1MTrp entities (Table 3). Both isomers of 1MTrp showed rapid washout from the blood, renal excretion, and low *in vivo* accumulation in almost all of the organs in normal rats. These findings can explain the absence of noticeable toxicity in preclinical and clinical antitumor studies with l-1MTrp, d-1MTrp, and a racemic mixture of these two isomers. Using PET imaging and an *ex vivo* biodistribution study, we directly visualized and confirmed markedly different in vivo distributions and behaviors of the l and d isomers (Figs 2, 3 and 4 and Tables 1 and 2). l-1MTrp exhibited the highest accumulation in the pancreas (SUV 2.28 and 2.27% ID/g, at 60 min), while d-1MTrp exhibited the highest retention in the kidney (SUV 1.79 and 1.16% ID/g, at 60 min). Absolute radioactivity levels of the two isomers in other organs and tissues were also obtained and quantified over time, although their concentrations were very low because of IDO silent in most normal tissues. Using these radiolabeled isomers in PET will provide objective evidence and allow determination of dose indexes for each organ, which is an important factor for elucidating the effects of 1MTrp *in vivo,* and will guide optimal dose selection and administration protocol design in development of 1MTrp immunotherapy.

l and d stereoisomers of 1MTrp, as specific IDO inhibitors, have been verified by a series of elegant studies with IDO-specific siRNA cells and IDO-deficient mice^5^,^15^. IDO is only detectable in several tissues: spleen, lung, small intestine, epididymis and placenta in normal physiological condition. Its overexpression is found and induced in a wide variety of human tumors and activated immune cells such as dendritic cells and antigen-presenting cells^8^,^11^,^21^. Increased expression of IDO1 has been recognized as an independent predictor of poor prognosis^22^,^23^. Corresponding to the induced IDO overexpression in cancer patients, *in vivo* pharmacokinetic changes of 1MTrp has been validated when treated with 1MTrp, and combined with conventional chemotherapy^24^,^25^. In the current study, ^11^C-l-1MTrp showed excellent pancreas imaging, with a good signal-to-noise ratio (Fig. 2), which seems to be associated with high expression of the LAT system that recognizes l-amino acids and a high rate of protein synthesis^26^. ^11^C-d-1MTrp also exhibited good *in vivo* sensitivity as a useful PET probe with rapid circulation clearance and fast tissue penetration (Fig. 2).

Thus, it is rational to state that use of ^11^C-l-1MTrp and ^11^C-d-1MTrp in PET represents a powerful platform for imaging IDO variation, monitoring the pharmacokinetic response to 1-MTrp, and identifying the relationship between the two isomers and antitumor effects, in a whole-body and temporal−spatial fashion. This study forms the basis for disentangling the effects of the two isomers of 1MTrp and suggests ways of stratifying a clinical subpopulation and deciding to continue or cease 1MTrp immunotherapy. We are currently exploring a means to determine the imaging potential of the 1MTrp pharmacokinetic platform in tumor-bearing models and in cancer immunotherapy; preliminary data have indicated great potential for the use of these compounds to detect IDO-positive tumors and as pharmacodynamics indicators with remarkable specificity and sensitivity.

## Conclusions

In this study, we have successfully developed ^11^C-l-1MTrp and ^11^C-d-1MTrp as two novel radioprobes for pharmacokinetic imaging of the checkpoint inhibitor 1MTrp. ^11^C-l-1MTrp and ^11^C-d-1MTrp were synthesized rapidly, with reliable radiochemical yield and sufficient radioactivity for evaluation. PET studies with ^11^C-l-1MTrp and ^11^C-d-1MTrp validated that the isomers have markedly different *in vivo* distributions and actions. This study forms the basis for disentangling the antitumor potential of the two isomers of 1MTrp and will facilitate the development of optimal 1MTrp immunotherapy.

## Methods and Materials

### General

All reagents were purchased from Sigma−Aldrich (St Louis, MO) and Wako Pure Industries (Osaka, Japan). 1-Methyl-l-tryptophan (l-1MTrp) and 1-Methyl-d-tryptophan (d-1MTrp) were obtained from Sigma−Aldrich. Radioactive carbon-11 (^11^C) was produced by a ^14^N (p, α) ^11^C nuclear reaction using a CYPRIS HM-18 cyclotron (Sumitomo Heavy Industry, Tokyo, Japan). Radioactivity was measured with an IGC-3R Curiemeter (Aloka, Tokyo). HPLC separation was performed using a JASCO HPLC system (JASCO, Tokyo): effluent radioactivity was determined using a NaI (Tl) scintillation detector system.

### Synthesis of Boc-Trp-OEt precursors for radiosynthesis

Two Boc-Trp-OEt precursors were prepared by reaction of *N*-Boc-l-Trp or *N*-Boc-d-Trp with bromoethane using NaOH as a base, as previously reported^27^. The enantiomeric purity of the two precursors was analyzed using a CHIRALPAK AS-RH column (4.6 mm i.d. × 150 mm; Daicel, Osaka, Japan) and CH_3_CN/H_2_O (40/60, v/v) at a flow rate of 1 mL/min and UV 225 nm. The retention times of *N*-Boc-d-Trp-OEt and *N*-Boc-l-Trp-OEt were 12.3 and 13.1 min, respectively. The enantiomeric purity of the two isomers was >80% ee.

### Radiosynthesis of ^11^C-l-1MTrp and ^11^C-d-1MTrp

^11^C-CH_3_I was synthesized from cyclotron-produced ^11^C-CO_2_, using an in-house developed automated synthesis system^28^,^29^. The produced ^11^C-CH_3_I was trapped in a mixture of the corresponding Boc-Trp-OEt precursor (0.9–1.1 mg) and NaOH (10 mg) in anhydrous DMSO (0.3 mL) at room temperature. The reaction mixture was heated at 80 °C for 5 min. Subsequently, the Boc and ethyl groups were deprotected using HCl (2 M, 0.5 mL) at 100 °C for 5 min. The reaction was terminated using 1.5 mL of the HPLC solvent; then, the radioactive mixture was loaded onto a preparative HPLC system for separation. HPLC separation was performed using a Capcell Pak UG 80 column (10 mm i.d. × 250 mm; Shiseido, Tokyo) and 20 mm phosphate buffer/CH_3_CN (8.5/1.5, v/v) as a mobile phase, at a flow rate of 5 mL/min. The fraction corresponding to ^11^C-l-1MTrp or ^11^C-d-1MTrp (*t*_R_: 9 min) was collected into a flask to which 25% ascorbic acid (0.4 mL) and Tween-80 (0.075 mL) in ethanol (0.3 mL) had been added prior to radiosynthesis and then evaporated to dryness. The residue was dissolved in distilled water (7 mL) and sterilized through a Millex-GS filter (Millipore, Billerica, MA) to obtain the final product. Radiochemical purity was assayed by analytical HPLC (column: Capcell Pack UG, 10 mm i.d. × 250 mm; UV: 225 nm; mobile phase: 20 mm phosphate buffer/CH_3_CN (8.5/1.5, v/v); flow rate: 1 mL/min). The t_R_ for ^11^C-l-1MTrp and ^11^C-d-1MTrp was 8.1 min. The identity of ^11^C-l-1MTrp and ^11^C-d-1MTrp was confirmed by co-injection with the corresponding unlabeled l-1MTrp or d-1MTrp on the analytical HPLC. The specific activity was measured and calculated by comparing the assayed radioactivity to the mass measured at UV on 225 nm. The enantiomeric purity of ^11^C-l-1MTrp and ^11^C-d-1MTrp was assayed using chiral analytical HPLC (column: CHIRALPAK ZWIX (+), 4 mm i.d. × 250 mm, Daicel; UV at 225 nm; mobile phase: CH_3_OH/H_2_O/HCOOH/Et_2_NH [500/10/0.945/1.295 v/v/v/v], at a flow rate of 1 mL/min). The t_R_ for ^11^C-d-1MTrp and ^11^C-l-1MTrp was 7.8 min and 9.4 min, respectively.

### Animals

Seven-week-old male Sprague-Dawley (SD) rats were obtained from Japan SLC (Shizuoka, Japan). The animals were maintained and handled in accordance with the recommendations of National Institute of Health and the institutional guidelines of National Institute of Radiological Sciences. The study protocols were approved by the Animal Ethics Committee of National Institute of Radiological Sciences. To ensure stable plasma tryptophan and other amino acid levels during the study, before all experiments, the rats were fasted overnight, while providing free access to water. All of the subsequent animal experiments were carried out with four rats in the respective groups.

### *In vitro* stability assay in rat plasma

^11^C-l-1MTrp and ^11^C-d-1MTrp (70–74 MBq, 0.4−0.6 nmol) was dissolved in 2 mL of freshly isolated rat plasma and incubated at 37 °C for 90 min. After 0, 30, 60 and 90 min, an aliquot of 300 μL incubated mixture was taken, proteins in plasma were precipitated with equal volumes of 1 M HClO_4_ solution, followed by centrifugation at 15000 rpm for 2 min at 4 °C. The supernatant was collected and analyzed by HPLC (Capcell Pack UG80 column, 4.6 mm i.d. × 250 mm) with 20 mm phosphate buffer/CH_3_CN (8.5/1.5, v/v) at a flow rate of 1 mL/min.

### Whole-Body PET/CT Scans

Whole-body PET scans were performed using a small-animal Siemens Inveon PET scanner (Siemens, Knoxville, TN) after intravenous injection of ^11^C-l-1MTrp or ^11^C-d-1MTrp (35–41 MBq/0.2 mL, 0.2−0.3 nmol), which provides 159 transaxial slices with 0.796-mm (center-to-center) spacing, a 10-cm transaxial field of view (FOV), and a 12.7-cm axial FOV. Emission scans were acquired in three-dimensional list mode with an energy window of 350–650 keV under isoflurane anesthesia, from 0 to 60 min after radioprobe administration. All list-mode acquisition data were sorted into three-dimensional sinograms, which were then Fourier-rebinned into two-dimensional sonograms (frames × min: 4 × 1, 8 × 2, 8 × 5), and corrections for scanner dead time, randoms, and decay of the injected radioprobe. Dynamic images were reconstructed with filtered back-projection using a Hanning filter and a Nyquist cut-off of 0.5 cycles/pixel. Immediately after completion of PET scans, contrast-enhanced CT scans were performed in the rats by using the small-animal CT system (R_mCT2; Rigaku, Tokyo) after injection of 1 mL of non-ionic contrast medium (Iopamiron 370, Bayer, Osaka). The scan conditions included radiation parameters of 200 μA and 90 kV, FOV of 60 mm, and acquisition time of 34 s. CT images were collected, reconstructed, and observed using I-View-R software (Rigaku, The Woodlands, TX). Averaged CT attenuation images and dynamic PET images were reconstructed and fused using the Siemens Inveon Research Workplace (IRW) 4.0 software.

Regions of interest (ROI) in major organs and tissues were drawn in the PET/CT fusion images by manual outlining the organs and tissues in the CT images throughout the image slices by using IRW 4.0 software. The average radioactivity concentration was obtained from mean pixel values in the ROI volume. Regional uptake of radioactivity was decay-corrected to the injection time and expressed as the standardized uptake value (SUV), normalized for injected radioactivity and body weight. SUV was calculated as SUV = (radioactivity per cubic centimeter tissue/injected radioactivity) × body weight in grams. TACs of the radioprobes in individual organ and tissue were determined. Values of area under time-activity curves (AUC_2–60 min_, SUV × min) were calculated from 2 to 60 min post-injection to minimize the early perfusion effects.

### *Ex vivo* biodistribution studies

After 17−18.5 MBq (0.1−0.15 nmol) of ^11^C-l-1MTrp or ^11^C-d-1MTrp injection, SD rats were sacrificed by cervical dislocation at five time points (1, 5, 15, 30, and 60 min). Major organs and tissues (blood, heart, lung, liver, pancreas, spleen, kidney, intestine, muscle, and brain), were promptly excised, harvested, and weighed. Radioactivity was counted using a γ-counter and expressed as a percentage of the injected dose per gram of wet tissue (%ID/g). All radioactivity measurements were corrected for decay.

### *Ex vivo* metabolite studies

SD rats were injected with ^11^C-l-1MTrp or ^11^C-d-1MTrp (70–74 MBq, 0.4–0.6 nmol) via a lateral tail vein. The animals were sacrificed by cervical dislocation at 15 and 60 min after the injection. Blood was collected and centrifuged at 15000 rpm for 2 min at 4 °C to separate the plasma. Brain and pancreas were rapidly removed, then homogenized in equal volumes of 1 M HClO_4_ solution with a Silent Crusher S homogenizer (Heidolph Instruments). Plasma and homogenates of brain and pancreas from rats were deproteinized and then analyzed by HPLC as described in the stability assay *in vitro*.

### Statistical analysis

All data are presented as the mean of the values ± the standard error of mean (SEM). Student’s *t*-test was used to compare the tissue distribution of the two isomers *in vivo*. The threshold for statistical significance was set at *p* < 0.05. All animal experimental results included data from four rats.

## Additional Information

**How to cite this article**: Xie, L. *et al.* Development of 1-*N*-^11^C-Methyl-l- and -d-Tryptophan for pharmacokinetic imaging of the immune checkpoint inhibitor 1-Methyl-Tryptophan. *Sci. Rep.* **5**, 16417; doi: 10.1038/srep16417 (2015).

## Figures and Tables

**Table 1:** Biodistribution of ^11^C-l-1MTrp in SD rats.

Tissue	1 min	5 min	15 min	30 min	60 min
Blood	1.34 ± 0.03	0.65 ± 0.01	0.54 ± 0.02	0.50 ± 0.01	0.50 ± 0.02
Heart	1.13 ± 0.01	0.64 ± 0.01	0.54 ± 0.02	0.49 ± 0.01	0.47 ± 0.02
Lung	0.98 ± 0.02	0.62 ± 0.01	0.50 ± 0.02	0.45 ± 0.01	0.43 ± 0.02
Liver	1.51 ± 0.03	0.73 ± 0.01	0.61 ± 0.03	0.55 ± 0.01	0.54 ± 0.02
Pancreas	2.95 ± 0.23	3.30 ± 0.12	2.65 ± 0.16	2.70 ± 0.17	2.27 ± 0.05
Spleen	0.98 ± 0.05	0.61 ± 0.00	0.55 ± 0.02	0.51 ± 0.01	0.48 ± 0.01
Kidney	2.60 ± 0.02	1.64 ± 0.03	1.11 ± 0.06	0.86 ± 0.03	0.74 ± 0.04
Intestine	1.27 ± 0.05	0.68 ± 0.01	0.53 ± 0.02	0.51 ± 0.00	0.46 ± 0.02
Muscle	0.18 ± 0.02	0.46 ± 0.01	0.46 ± 0.02	0.45 ± 0.01	0.45 ± 0.02
Brain	0.14 ± 0.00	0.20 ± 0.01	0.27 ± 0.02	0.30 ± 0.02	0.31 ± 0.01

Data are %ID/g tissue (mean ± SEM; n = 4).

**Table 2:** Biodistribution of ^11^C-d-1MTrp in SD rats.

Tissue	1 min	5 min	15 min	30 min	60 min
Blood	1.47 ± 0.02	0.79 ± 0.03	0.49 ± 0.01	0.40 ± 0.01	0.32 ± 0.01
Heart	0.58 ± 0.02	0.55 ± 0.02	0.52 ± 0.02	0.42 ± 0.02	0.30 ± 0.01
Lung	0.79 ± 0.03	0.60 ± 0.02	0.46 ± 0.02	0.37 ± 0.01	0.28 ± 0.01
Liver	1.24 ± 0.03	0.97 ± 0.02	0.64 ± 0.01	0.47 ± 0.01	0.37 ± 0.01
Pancreas	0.76 ± 0.07	1.03 ± 0.15	1.01 ± 0.06	1.21 ± 0.05	1.03 ± 0.06
Spleen	0.62 ± 0.02	0.51 ± 0.02	0.48 ± 0.01	0.40 ± 0.02	0.32 ± 0.01
Kidney	7.06 ± 0.57	6.21 ± 0.64	3.66 ± 0.15	2.12 ± 0.16	1.16 ± 0.03
Intestine	0.93 ± 0.02	0.68 ± 0.02	0.53 ± 0.00	0.40 ± 0.01	0.32 ± 0.01
Muscle	0.12 ± 0.00	0.26 ± 0.01	0.35 ± 0.01	0.38 ± 0.03	0.33 ± 0.01
Brain	0.04 ± 0.00	0.05 ± 0.01	0.08 ± 0.00	0.11 ± 0.00	0.15 ± 0.01

Data are %ID/g tissue (mean ± SEM; n = 4).

**Table 3:** *Ex vivo* metabolite analysis of ^11^C-l-1MTrp and ^11^C-d-1-MTrp in the plasma, brain and pancreas of SD rats.

Timeafterinjection	^11^C-l-1MTrp			^11^C-d-1-MTrp		
	Plasma	Brain	Pancreas	Plasma	Brain	Pancreas
15 min	97.67 ± 0.18	98.38 ± 0.34	94.73 ± 1.64	94.45 ± 0.03	95.06 ± 0.38	94.08 ± 0.51
60 min	98.44 ± 0.13	98.93 ± 0.51	95.88 ± 0.64	98.81 ± 0.25	98.16 ± 0.47	96.31 ± 0.48

Data are expressed as % of tailored l- and d-1MTrp entity (mean ± SEM; n = 4).
